# The Role of Fluorine-18-Fluorodeoxyglucose Positron Emission Tomography/Computed Tomography (¹⁸F-FDG PET/CT) in Staging and Treatment Response Assessment of Metastatic Melanoma: A Case Report

**DOI:** 10.7759/cureus.103160

**Published:** 2026-02-07

**Authors:** David Gutiérrez, Ana María Gutiérrez, Mariana Parra, Ian Taylor, Gabriel Infante

**Affiliations:** 1 Cyclotron-Positron Emission Tomography/Computed Tomography, University of Costa Rica, San José, CRI; 2 General Medicine, University of Costa Rica, San José, CRI

**Keywords:** assessment, computed tomography, fluorine-18-fluorodeoxyglucose, metastatic melanoma, positron emission tomography, staging

## Abstract

Melanoma is a malignant neoplasm with an increasing incidence in the last few years. It has a great metastatic capacity with an unpredictable pattern of spread. For evaluating the extent of the disease and identifying and locating metastases, fluorine-18-fluorodeoxyglucose (FDG) positron emission tomography (PET) combined with computed tomography (CT) should be considered mandatory at baseline for tumor assessment before the start of immunotherapy.

This report describes the case of a 53-year-old male diagnosed with stage IV malignant melanoma presenting with multiple metastatic lesions who underwent immuno- and radiotherapy. It acknowledges the relevant role of FDG PET/CT in the staging and assessment of progress and response to treatment in patients with melanoma, due to the combination of metabolic (PET) and anatomical (CT) images.

## Introduction

Melanoma is a cutaneous malignancy that has shown a rapid increase in incidence in the last few years. Its high metastatic potential and unpredictable dissemination pattern remain the main causes of mortality among affected patients [1,2]. Nowadays, standard treatment of advanced malignant melanoma is based on immune checkpoint inhibitors (ICI), such as ipilimumab and nivolumab. These treatments have shown a significant improvement in the survival rate. However, a significant number of patients do not respond to therapy and may experience immune-related adverse events (irAE) [3,4].

Early identification of patients unlikely to benefit from immunotherapy, as well as prompt recognition of irAEs, is crucial for guiding treatment decisions and improving prognosis [5]. Objective and reliable tumor assessment, therefore, plays a key role in patient management. According to the European Association of Nuclear Medicine (EANM), the Society of Nuclear Medicine and Molecular Imaging (SNMMI), and the Australian and New Zealand Society of Nuclear Medicine (ANZSNM) guidelines, fluorine-18-fluorodeoxyglucose (FDG) positron emission tomography (PET) combined with computed tomography (CT) should be considered mandatory at baseline for tumor assessment before the start of immunotherapy [6]. Based on the National Comprehensive Cancer Network (NCCN) guidelines, routine cross-sectional imaging is not recommended for patients in stage 0, I, or IIA but should be considered every 3 to 12 months for stage IIB-IV patients (evidence level 2B) [7].

Melanoma cells usually exhibit avid uptake of the glucose analog FDG, reflecting the intracellular glucose metabolism and providing information about tissue metabolism; therefore, a functional imaging method is recommended [8]. Whole-body FDG PET/CT is the most used imaging method in patients with high-risk melanoma for tumor staging, prognostication, and assessment of therapy response. This method integrates metabolic (PET) and anatomical (CT) images and allows more accurate determination of abnormal sites. Being the most frequent sites of metastasis, the lungs, lymph nodes, and muscle tissue [2,9-11].

This case highlights the pivotal role of whole-body FDG PET/CT in the comprehensive evaluation of high-risk melanoma, illustrating its utility for disease staging, monitoring therapeutic response, and assessing clinical evolution.

## Case presentation

A 53-year-old male with no significant past medical history was referred to a tertiary care center with a diagnosis of stage IV malignant melanoma, presenting with metastatic involvement of the brain, mediastinum, lungs, peritoneum, and para-aortic and inguinal lymph nodes. No primary cutaneous lesion was identified. At the time of referral, the patient was receiving combined immunotherapy with ipilimumab and nivolumab and had undergone two sessions of brain-directed radiotherapy, with two additional sessions planned. A whole-body ¹⁸F-FDG PET/CT scan was requested for disease staging. 

The baseline PET/CT revealed extensive hypermetabolic disease consistent with widespread metastases. At the head and neck level, at least four foci of increased FDG uptake were identified within the brain parenchyma, compatible with neoplastic infiltration, along with bilateral cervical lymphadenopathy involving levels II and III. Thoracic findings included a hypermetabolic mass with irregular margins at the left lung base and hypermetabolic subcarinal and left parahilar lymph nodes. In the abdomen and pelvis, multiple hypermetabolic lymph nodes were detected in the para-aortic, pre-aortic, interaortocaval, and precaval regions. Additionally, at least five hypermetabolic peritoneal nodules consistent with neoplastic implants were observed, as well as focal uptake corresponding to the fourth portion of the duodenum. Musculoskeletal involvement was evidenced by multiple hypermetabolic nodular soft-tissue lesions in the right upper thoracic region, right deep lumbar area, and left lateral thigh, and a nodular lesion within the lateral head of the left gastrocnemius muscle, all suggestive of metastatic infiltrations. Baseline ¹⁸F-FDG PET/CT revealed extensive hypermetabolic metastatic disease involving the brain, nodal stations, lung base, peritoneum, and multiple soft-tissue sites, with SUVmax values ranging from 4.5 to 19.8, consistent with widespread neoplastic infiltration.

**Figure 1:**
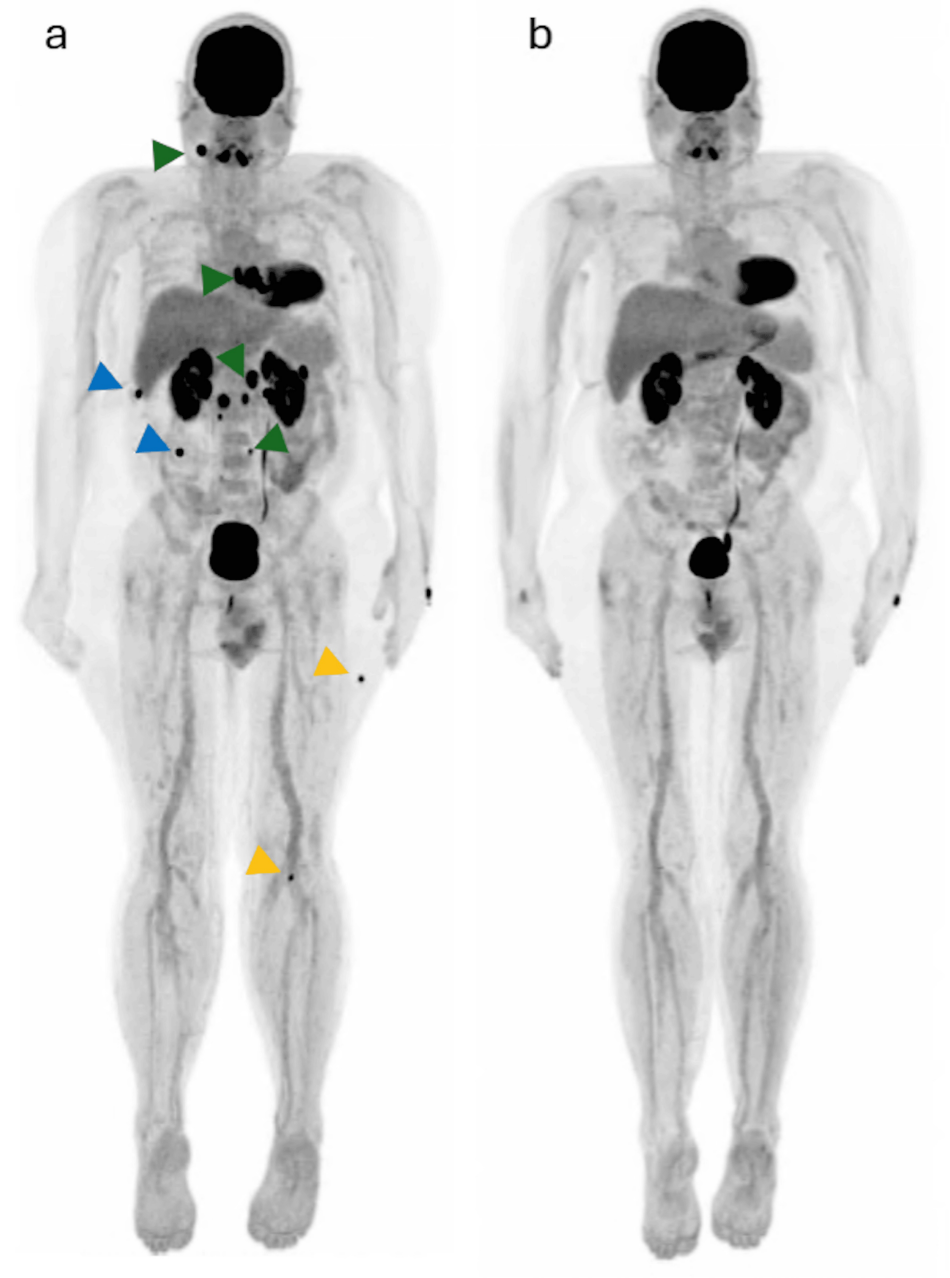
a) Maximum intensity projection (MIP) image demonstrating FDG-avid cervical and retroperitoneal lymph nodes (green arrowheads), peritoneal implants (blue arrowheads), and muscle lesions (yellow arrowheads). b) Follow-up MIP image obtained three months after the initial FDG-PET/CT, showing a marked reduction in FDG uptake with a near-complete metabolic response.

**Figure 2:**
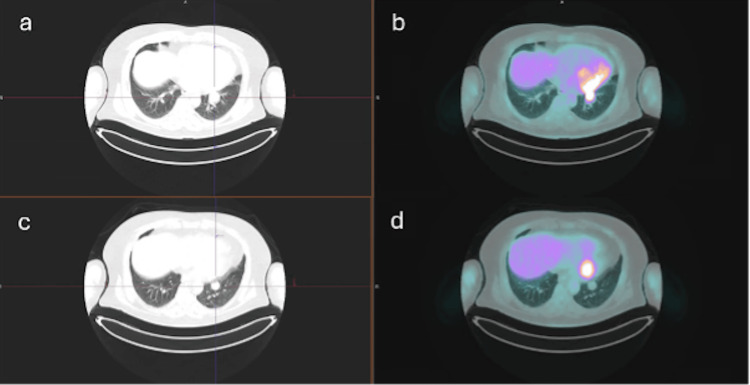
Axial CT and axial FDG-PET/CT images (a, b) demonstrating a hypermetabolic left basal pulmonary nodule, consistent with metastatic disease. Follow-up axial CT and axial FDG-PET/CT images obtained 3 months after the initial FDG-PET/CT (c, d) show a marked reduction in FDG uptake, with a near-complete metabolic response comparable to background activity.

After 11 weeks, while the patient continued immunotherapy and following completion of four sessions of brain radiotherapy, a follow-up ¹⁸F-FDG PET/CT scan was performed using identical acquisition parameters. As shown in Figures 1, 2, the study demonstrated a complete metabolic response in all previously described lesions, with only minimal residual FDG uptake in a left pulmonary nodule and a subcarinal lymph node, below reference organ activity and likely related to post-therapeutic or inflammatory changes rather than residual viable tumor.

## Discussion

Melanoma is a highly metastatic disease. Imaging has an essential role in the identification, localization, and characterization of metastases and, therefore, in the management and prognosis of melanoma. Conventional imaging techniques, like radiography, ultrasonography, computed tomography, and magnetic resonance, are of limited value in identifying melanoma metastases [2]. Several systematic reviews and meta-analyses have demonstrated the superior diagnostic performance of ¹⁸F-FDG PET/CT compared with conventional imaging modalities for the detection of distant melanoma metastases, particularly in advanced-stage disease [2,5].

The patient presented with a confirmed diagnosis of metastatic malignant melanoma; however, the full extent and distribution of metastatic disease had not been previously established. The initial staging ¹⁸F-FDG PET/CT was performed after initiation of combined immunotherapy with ipilimumab and nivolumab, at a time when the exact duration of treatment was unknown. The widespread distribution of metastatic lesions observed in our patient, including lymphatic, pulmonary, peritoneal, muscular, and central nervous system involvement, is consistent with the heterogeneous metastatic patterns described in large imaging series of advanced melanoma [10]. Although current EANM/SNMMI/ANZSNM guidelines recommend performing baseline ¹⁸F-FDG PET/CT prior to the initiation of immunotherapy, the study remained clinically valuable by accurately delineating the extent of disease involvement.

In terms of literature, the PET alone does not recognize lesions in the lung, but in combination with CT, the sensitivity increases [2,9,10]. Also, the combined use of both modalities shows an improved role in the detection and differentiation of distant metastases, especially of visceral metastases [9]. 

There is no consensus in timing between imaging for decision-making and treatment management [5,12]. Regarding treatment response assessment, previous studies evaluating patients treated with immune checkpoint inhibitors have shown that early metabolic response on ¹⁸FDG PET/CT correlates with improved clinical outcomes and overall survival, even when performed within the first 2-3 months of therapy. Our patient demonstrated a near-complete metabolic response on follow-up ¹⁸FDG PET/CT performed 11 weeks after baseline imaging, a finding that correlates with published evidence supporting the prognostic value of metabolic response in melanoma patients undergoing immunotherapy [5,12]. The findings illustrate the ability of ¹⁸F-FDG PET/CT to provide early and reliable assessment of therapeutic efficacy. 

A limitation presented in the case was the absence of a true baseline FDG-PET/CT before the initiation of treatment, so we could not ensure that there weren't any other initial lesions, and we could not describe the progress after beginning treatment with ipilimumab, nivolumab, and radiotherapy.

While most guidelines recommend obtaining a baseline ¹⁸FDG PET/CT prior to the initiation of immunotherapy, several reviews acknowledge that PET/CT performed after treatment initiation can still provide clinically relevant information regarding disease extent and therapeutic efficacy [5]. In this context, our case reinforces the role of ¹⁸FDG PET/CT as a robust tool for both staging and response assessment, even when a true pretreatment baseline is unavailable. 

## Conclusions

Melanoma has a great metastatic capacity. Therefore, appropriate imaging studies should be used for staging the disease and identifying the location of metastases. Whole-body ¹⁸F-FDG PET/CT is the preferred imaging modality in advanced melanoma due to its high sensitivity and its ability to integrate metabolic and anatomical information. In the presented current case, an adult man with metastatic melanoma, with the aid of FDG-PET/CT, we were able to stage the disease and subsequently establish that all the lesions previously described had a complete metabolic response after finishing radiotherapy and while completing immunotherapy.

While a single descriptive case cannot establish causality or define mandatory clinical pathways, our findings are consistent with existing evidence from systematic reviews and current international guidelines that support the use of ¹⁸FDG PET/CT as a key imaging modality in advanced-stage melanoma. Nevertheless, this case reinforces the role of ¹⁸FDG PET/CT as a valuable imaging tool for staging and treatment monitoring in metastatic melanoma.
